# From Vision to Illumination: The Promethean Journey of Optical Coherence Tomography in Cardiology

**DOI:** 10.3390/jcm14155451

**Published:** 2025-08-02

**Authors:** Angela Buonpane, Giancarlo Trimarchi, Francesca Maria Di Muro, Giulia Nardi, Marco Ciardetti, Michele Alessandro Coceani, Luigi Emilio Pastormerlo, Umberto Paradossi, Sergio Berti, Carlo Trani, Giovanna Liuzzo, Italo Porto, Antonio Maria Leone, Filippo Crea, Francesco Burzotta, Rocco Vergallo, Alberto Ranieri De Caterina

**Affiliations:** 1 Center of Excellence for Cardiovascular Sciences, Isola Tiberina-Gemelli Isola Hospital, 00186 Rome, Italy; antoniomaria.leone@fbf-isola.it (A.M.L.); filippo.crea@fbf-isola.it (F.C.); 2 Cardiothoracic Department, Fondazione Toscana G. Monasterio, G. Pasquinucci Heart Hospital, 54100 Massa, Italy; giancarlo.trimarchi18@gmail.com (G.T.); mciard@ftgm.it (M.C.); michecoc@ftgm.it (M.A.C.); lpastor@ftgm.it (L.E.P.); uparadossi@ftgm.it (U.P.); ifcberti@ftgm.it (S.B.); 3 Interdisciplinary Center for Health Sciences, Scuola Superiore Sant’Anna, 56100 Pisa, Italy; 4 Department of Medicine, Surgery and Dentistry, University of Salerno, 84081 Salerno, Italy; fdimuro94@gmail.com; 5 Department of Cardiothoracic and Vascular Sciences, Careggi University Hospital, 50124 Firenze, Italy; giulia.nardi.fi@gmail.com; 6 Department of Cardiovascular Sciences, Fondazione Policlinico Universitario A. Gemelli, Scientific Institute for Research, Hospitalization and Healthcare, Università Cattolica Sacro Cuore, 00168 Rome, Italy; carlo.trani@unicatt.it (C.T.); giovanna.liuzzo@unicatt.it (G.L.); francesco.burzotta@unicatt.it (F.B.); 7 Scientific Institute for Research, Hospitalization and Healthcare, Ospedale Policlinico San Martino, Università di Genova, 16162 Genoa, Italy; italo.porto@unige.it (I.P.); rocco.vergallo@unige.it (R.V.)

**Keywords:** Optical Coherence Tomography, coronary artery disease, intravascular imaging

## Abstract

Optical Coherence Tomography (OCT) has evolved from a breakthrough ophthalmologic imaging tool into a cornerstone technology in interventional cardiology. After its initial applications in retinal imaging in the early 1990s, OCT was subsequently envisioned for cardiovascular use. In 1995, its ability to visualize atherosclerotic plaques was demonstrated in an in vitro study, and the following year marked the acquisition of the first in vivo OCT image of a human coronary artery. A major milestone followed in 2000, with the first intracoronary imaging in a living patient using time-domain OCT. However, the real inflection point came in 2006 with the advent of frequency-domain OCT, which dramatically improved acquisition speed and image quality, enabling safe and routine imaging in the catheterization lab. With the advent of high-resolution, second-generation frequency-domain systems, OCT has become clinically practical and widely adopted in catheterization laboratories. OCT progressively entered interventional cardiology, first proving its safety and feasibility, then demonstrating superiority over angiography alone in guiding percutaneous coronary interventions and improving outcomes. Today, it plays a central role not only in clinical practice but also in cardiovascular research, enabling precise assessment of plaque biology and response to therapy. With the advent of artificial intelligence and hybrid imaging systems, OCT is now evolving into a true precision-medicine tool—one that not only guides today’s therapies but also opens new frontiers for discovery, with vast potential still waiting to be explored. Tracing its historical evolution from ophthalmology to cardiology, this narrative review highlights the key technological milestones, clinical insights, and future perspectives that position OCT as an indispensable modality in contemporary interventional cardiology. As a guiding thread, the myth of Prometheus is used to symbolize the evolution of OCT—from its illuminating beginnings in ophthalmology to its transformative role in cardiology—as a metaphor for how light, innovation, and knowledge can reveal what was once hidden and redefine clinical practice.

## 1. Introduction

*“If I have seen further, it is by standing on the shoulders of giants”.*

(Isaac Newton, letter to Robert Hooke, 5 February 1675)

*This work is dedicated to all the researchers upon whose shoulders we stand.*

In ancient myth, Prometheus defied the gods to steal fire and deliver it to humankind, igniting the dawn of civilization. In modern medicine, a similar leap occurred—not from myth, but from the convergence of physics and biology: Optical Coherence Tomography (OCT) has introduced a new form of vision, capable of imaging biological tissues in vivo with micrometer-scale resolution and in real time. Initially developed for ophthalmology, OCT has found one of its most impactful applications in interventional cardiology. By enabling high-resolution visualization of coronary arteries from within, it has redefined the diagnosis and treatment of coronary artery disease (CAD). OCT provides the ability to guide and optimize every stage of percutaneous coronary intervention (PCI), significantly improving procedural precision and reducing complications, repeat revascularizations, and cardiovascular mortality. Beyond its clinical utility, OCT has also become a powerful tool for advancing our understanding of atherosclerosis. Its unparalleled resolution has allowed for detailed characterization of plaque morphology, identification of mechanisms of plaque destabilization, and longitudinal assessment of disease progression in vivo. In this respect, OCT serves not only as a diagnostic and therapeutic adjunct but also as a unique platform for scientific research, bridging imaging with pathophysiology in ways previously unattainable.

**Figure 1:**
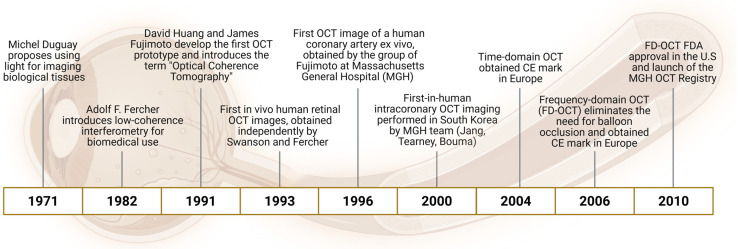
Key milestones in the development of Optical Coherence Tomography (OCT): this timeline highlights the major scientific and technological advances that led to the development and clinical adoption of OCT. From the initial concept of using light for biological imaging in the 1970s to the introduction of low-coherence interferometry and the first in vivo human coronary imaging. The evolution from time-domain to frequency-domain OCT enabled real-time intracoronary imaging without balloon occlusion, paving the way for widespread clinical use and regulatory approval.

This narrative review traces the evolution of OCT—from its origins in optical physics to its integration into daily cardiology practice—and highlights how this technology has reshaped not only interventional strategies but also our broader understanding of coronary atherosclerosis (Figure 1). To ensure a comprehensive and up-to-date overview, we performed a non-systematic literature search in PubMed/MEDLINE and ClinicalTrials.gov, using terms including “optical coherence tomography,” “atherosclerosis,” “plaque erosion,” “OCT-guided PCI,” and “coronary imaging.” Particular emphasis was placed on randomized controlled trials, high-impact observational studies, and major consensus documents published in the past 15 years. Selection and interpretation were conducted independently by two reviewers to mitigate selection bias and maintain narrative rigor, in accordance with SANRA (Scale for the Assessment of Narrative Review Articles) recommendations.

## 2. From Light to Insight: The Origins of Optical Coherence Tomography

The conceptual roots of OCT trace back to 1971, when physicist Michel Duguay, working at Bell Labs, proposed a bold idea: that light, like sound in ultrasound, could be used to visualize internal structures within biological tissues. His work with ultrafast lasers suggested that scattered photons might one day reconstruct images of otherwise inaccessible anatomy. However, the technological limitations of the time—particularly the lack of detectors capable of resolving such short time scales—kept the concept theoretical for more than a decade [1].

A foundational step occurred in the early 1980s, when physicist Adolf Friedrich Fercher, a visionary in biomedical optics, began applying partial coherence interferometry (PCI) to biological systems. In 1982, Fercher proposed and patented a method for measuring intraocular distances using low-coherence interferometry. Working initially at the University of Essen and later at the Medical University of Vienna, his group demonstrated that micrometer-scale resolution could be achieved in vivo using this technique—laying the physical groundwork for what would become time-domain OCT [2].

While Fercher advanced the clinical potential of PCI in Europe, a parallel thread of development was unfolding at the Massachusetts Institute of Technology (MIT), where physicists Erich Ippen and James Fujimoto were at the forefront of ultrafast optics. Ippen, a pioneer in femtosecond laser science, trained a generation of physicists who would later apply these techniques beyond fundamental physics. Among them, James Fujimoto began exploring the biomedical applications of ultrafast and low-coherence light sources. While femtosecond lasers offered unmatched temporal resolution, their bulkiness, high cost, and limited sensitivity rendered them impractical for clinical use. A turning point came through interdisciplinary collaboration: Fujimoto joined forces with Dr. Carmen Puliafito, a retinal specialist at the New England Eye Center, identifying ophthalmology as an ideal proving ground for innovative optical techniques. In 1991, David Huang, an MD-PhD student in Fujimoto’s lab, developed a scanning interferometric system capable of acquiring depth-resolved axial profiles (A-scans) and compiling them into cross-sectional images (B-scans). [3]. These first optical tomograms—recorded in vitro—were published in Science, marking the formal introduction of the term “Optical Coherence Tomography” and defining a new imaging modality [4].

Yet, those lasers were impractical for widespread use: large, expensive, and insufficiently sensitive for deep tissue imaging. Eric Swanson, an expert in optical communications from MIT Lincoln Lab, introduced the crucial element of interferometric amplification—enabling micrometer-scale detection of weak backscattered photons by synchronizing them with a reference wave. Early acquisitions took minutes, but with Swanson’s optimizations and a novel patient interface, they were able to accelerate image capture by 100-fold. Swanson implemented interferometric signal amplification and developed a stable scanning interface that enabled rapid acquisition of high-fidelity images. It was this technical sophistication—marrying optics with engineering—that transformed OCT from a laboratory prototype into a viable imaging modality [5].

The technology soon transitioned from in vitro experiments to in vivo imaging, with the first human retinal scans performed in 1993 at the New England Eye Center by Carmen Puliafito and Joel Schuman, capturing pathologies such as diabetic retinopathy and glaucoma [6]. What followed was a global race toward in vivo imaging. Fercher, immediately recognizing the clinical potential of this technology, focused his efforts on achieving OCT imaging in the living human eye. His group succeeded in doing so shortly after the MIT team: the first in vivo human retinal OCT image from Fercher’s lab was published in 1993, just a few weeks after the MIT group released their corresponding results [7]. Both teams had independently reached a critical milestone—the transition from optical bench to clinical applicability.

The impact was immediate. OCT rapidly became a standard in ophthalmology and was soon commercialized through a spin-off company that would later be acquired by Carl Zeiss Meditec. By 1996, the clinical utility of OCT was undeniable, leading to commercialization through a spin-off acquired by Zeiss. A technology born from quantum optics, refined through engineering, and guided by clinical necessity had entered the medical mainstream—first in ophthalmology, and eventually far beyond [5].

What began as a speculative idea in the minds of physicists became, through the contributions of researchers across disciplines and continents, one of the most transformative imaging technologies in clinical medicine. OCT was not the product of a single invention, but the convergence of visionary scientists, clinicians, and engineers—each bringing their own lens of expertise. From Fercher’s precision interferometry to Fujimoto’s optical insights, from Huang’s translational leap to Swanson’s technical rigor and Puliafito’s clinical foresight, the history of OCT exemplifies the power of interdisciplinary innovation.

## 3. From Time to Frequency: The Evolution of OCT Technology

The eye became the initial gateway for optical imaging technologies, which would later revolutionize diagnostic capabilities across medical disciplines. In 1993, the first in vivo images of the human retina were acquired using time-domain OCT (TD-OCT) at the New England Eye Center, marking a decisive clinical milestone [6]. These early systems, based on mechanically scanning a reference arm to obtain depth information, allowed noninvasive visualization of retinal microstructures at an axial resolution of approximately 10 μm. Despite their limited speed and sensitivity, TD-OCT enabled clinicians to detect and monitor conditions such as macular degeneration, diabetic retinopathy, and glaucoma—without contrast agents or ionizing radiation.

The real turning point in OCT performance, however, came not from clinical adaptation but from a fundamental advance in physics and signal processing: the transition to Fourier-Domain OCT (FD-OCT). Developed in the late 1990s and pioneered by researchers including Adolf Friedrich Fercher and colleagues in Vienna, FD-OCT abandoned mechanical delay scanning in favor of spectral detection. Instead of measuring echo delay sequentially, the entire backscattered interference spectrum was captured and processed using a Fourier transform to reconstruct depth profiles [8].

This shift brought about a dramatic increase in performance. FD-OCT improved signal-to-noise ratio by over 20 dB and increased imaging speed from a few hundred to tens of thousands of A-scans per second. These gains were not incremental—they were transformative. Faster acquisition eliminated motion artifacts and enabled high-resolution, three-dimensional reconstructions of the retina in near real time. Where TD-OCT offered static, cross-sectional slices, FD-OCT introduced dynamic volumetric imaging that allowed clinicians to visualize and quantify retinal pathology in unprecedented detail [9].

This technological leap transformed OCT from a specialized diagnostic instrument into an indispensable clinical tool. By the early 2000s, FD-OCT had entered routine ophthalmic practice and soon became the standard of care. In retrospect, the introduction of FD-OCT represents one of the most significant inflection points in the history of biomedical imaging. It did not merely enhance what was possible with OCT—it redefined the boundaries of noninvasive optical diagnostics. The ability to “see” the retina in motion, in three dimensions, and in vivo, reshaped not only ophthalmology but laid the foundation for OCT’s expansion into other fields, including cardiology, dermatology, and oncology.

## 4. Into the Heart: The Rise of Intravascular OCT (1998–2010)

At the dawn of the new millennium, OCT began to extend its transformative potential from ophthalmology to the field of interventional cardiology, marking a new era in intravascular imaging. Here, one of the earliest and most visionary contributors was Mark Brezinski, a physician-scientist working in the MIT laboratory of James Fujimoto. In 1996, Brezinski helped pioneer the concept of “optical biopsy,” demonstrating that Optical Coherence Tomography could resolve the microscopic architecture of atherosclerotic plaques in coronary arteries ex vivo, with unprecedented detail. His landmark studies showed that features such as thin fibrous caps, lipid pools, and macrophage infiltration—hallmarks of vulnerable plaque—could be visualized using OCT. The idea of employing OCT to identify plaque vulnerability had already been proposed by Brezinski and colleagues as early as 1995, laying the foundation for a new diagnostic paradigm. Notably, the first OCT image of a human coronary artery obtained ex vivo was published in 1996, depicting an unstable plaque characterized by a thin intimal cap adjacent to a heavily calcified area with minimal lipid content—an early but striking demonstration of OCT’s ability to capture complex plaque morphology with near-histological resolution. These findings were foundational: they proved that light could offer histology-like information without excision or staining [10]. In 1998, a pioneering collaboration formed at Massachusetts General Hospital and MIT, bringing together Dr. Ik-Kyung Jang, biomedical optics experts Brett Bouma and Guillermo Tearney, and a team determined to adapt OCT for intracoronary imaging. Their goal was audacious: to transform OCT—so successful in the transparent environment of the eye—into a tool capable of penetrating the opaque, blood-filled landscape of human arteries [11]. The conceptual leap involved replacing the ultrasound transducer in an intravascular ultrasound (IVUS) catheter with a single-mode optical fiber. But unlike sound waves, light is heavily scattered and absorbed by blood. To overcome this, the team engineered a system that temporarily flushed the coronary artery with saline or contrast to clear the optical path. The prototype required a balloon occlusion proximal to the imaging site to halt blood flow, a technically demanding and clinically limiting solution. Before moving to patients, the team conducted extensive validation studies. More than 350 human coronary artery specimens were analyzed ex vivo, demonstrating OCT’s ability to resolve microscopic structures—fibrous caps, macrophage clusters, lipid cores, and thrombi—with tenfold higher resolution than IVUS. While IVUS provided axial resolution of 100–150 μm, OCT revealed features on the 10 μm scale, approaching the granularity of histology [12].

This innovation culminated in 2000, when the team performed the first-in-human intracoronary OCT imaging in Republic of Korea [13]. The procedure was carried out in a 65-year-old man admitted for unstable angina pectoris. Coronary angiography revealed a critical stenosis in the right coronary artery, which was subsequently treated with implantation of a 3.0 × 16 mm Nanoporous Intraluminal Rod (NIR) stent. Post-PCI angiographic results were satisfactory, and IVUS confirmed good stent expansion and apposition. OCT imaging—performed with a 3.2 F catheter and a time-domain system—provided additional insights beyond those available with IVUS. Although the technique required balloon occlusion and saline flushing to clear blood and enable optical imaging, the results were groundbreaking. The OCT cross-sections not only confirmed proper stent apposition but also revealed tissue prolapse between the stent struts—a finding not detected by IVUS. This protruding tissue corresponded to an area of lower signal intensity, interpreted as lipid-rich plaque—suggestive of structural vulnerability. Retrospective analysis of the IVUS dataset hinted at the same feature but with far less clarity [13].

The first generation of commercial intracoronary OCT systems, based on TD-OCT, emerged shortly thereafter. These systems required a balloon occlusion and synchronized saline flush to obtain usable images—procedurally cumbersome and not easily generalizable. Although they received CE (Conformité Européenne) mark approval in Europe by 2004, the U.S. Food and Drug Administration (FDA) withheld approval, citing the complexity and invasiveness of the system. The real breakthrough arrived in 2006 with the development of FD-OCT [5]. This innovation eliminated the need for balloon occlusion, enabled automated pullback speeds up to 20 mm/s, and allowed the acquisition of >50 mm arterial segments in under 3 s. Imaging became simpler, faster, and safer, and with it came broader clinical adoption. In 2010, the FDA finally approved intracoronary FD-OCT for use in the United States [5]. That same year, the Massachusetts General Hospital (MGH) OCT Registry was launched, encompassing data from 21 centers in six countries, capturing thousands of clinical cases. OCT was no longer an experimental technique—it had become a frontline imaging modality in interventional cardiology [5]. It could now guide PCI in real time and reveal the living pathobiology of atherosclerosis. OCT had leapt from the eye to the heart, not by abandoning its roots, but by reimagining its principles in a radically different vascular environment.

## 5. The Clinical Evolution of OCT in Cardiology

### OCT-Guided PCI: From Feasibility to Superiority

**Figure 2:**
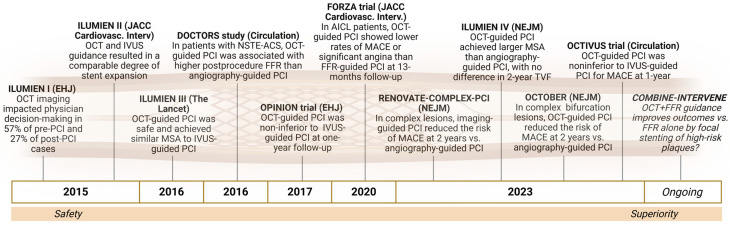
Key clinical trials supporting the integration of OCT in PCI practice: this timeline illustrates the progression from feasibility and safety to comparative efficacy and procedural superiority of OCT-guided PCI in both simple and complex coronary lesions. These studies consistently demonstrated the ability of OCT to improve decision-making, stent sizing, and optimization, leading to better anatomical and physiological outcomes.

The integration of OCT into PCI practice evolved through a series of landmark studies aimed at demonstrating its clinical utility and procedural safety (Figure 2).

The ILUMIEN I (Observational Study of Optical Coherence Tomography in Patients Undergoing Fractional Flow Reserve and Percutaneous Coronary Intervention) trial, a large prospective registry, was pivotal in showing that OCT guidance could influence decision-making during PCI. In over half of the cases, operators revised their interventional strategy after assessing pre-PCI plaque characteristics with OCT—often adjusting stent length and diameter. Even after what was deemed a successful stent implantation based on angiography, post-procedural OCT revealed issues such as underexpansion, malapposition, or edge dissections in more than a quarter of cases, prompting further optimization. These refinements translated into improved physiological outcomes, as measured by post-PCI fractional flow reserve (FFR), and low complication rates [14]. Building on this, ILUMIEN II offered a comparative lens by analyzing outcomes of OCT-guided PCI (from ILUMIEN I) against those guided by IVUS in the ADAPT-DES study [15]. Although the final minimal stent area (MSA) was similar between the two groups, OCT offered superior stent symmetry and more precise detection of underexpansion and malapposition—key predictors of restenosis and thrombosis. To validate these findings in a randomized setting, ILUMIEN III directly compared OCT-, IVUS-, and angiography-guided PCI [16]. The results confirmed that OCT-guided PCI achieved comparable stent expansion to IVUS, but with a more standardized methodology for stent sizing and optimization. Importantly, there were no significant differences in short-term clinical events among the three groups, supporting OCT’s non-inferiority and potential advantages in procedural precision.

In the DOCTORS (Does Optical Coherence Tomography Optimize Results of Stenting) trial, this anatomical superiority translated into improved physiology. Nearly half of the patients undergoing OCT-guided stenting required further optimization based on intra-procedural findings, such as underexpansion or edge dissection. These adjustments resulted in significantly higher post-PCI FFR, suggesting better functional revascularization [17]. The OPINION (Optical Frequency Domain Imaging vs. Intravascular Ultrasound in Percutaneous Coronary Intervention) trial further confirmed that OCT was non inferior to IVUS in terms of 12-month target vessel failure, reinforcing OCT’s role as a safe and effective imaging alternative [18]. The field then culminated in more ambitious studies. ILUMIEN IV, published in the New England Journal of Medicine in 2023, randomized nearly 2500 patients with either diabetes or complex coronary lesions to OCT- or angiography-guided PCI. The study found that OCT guidance achieved significantly larger MSA and reduced stent thrombosis rates. While target vessel failure at 2 years was not statistically different between groups, the trial validated the safety of OCT and standardized its use with defined pre- and post-stent optimization protocols [19]. However, it should be noted that ILUMIEN IV used primarily surrogate imaging endpoints, with relatively short-term clinical follow-up. Additionally, as a sponsor-driven trial, the possibility of design or reporting bias cannot be completely excluded. These factors must be considered when interpreting the strength and generalizability of its conclusions.

In recent years, the role of OCT has been rigorously tested in the setting of complex coronary lesions—a domain traditionally dominated by IVUS. Among the most influential studies in this space is RENOVATE-COMPLEX, a randomized controlled trial that enrolled patients with challenging anatomies, including unprotected left main disease, bifurcations, long or severely calcified lesions, chronic total occlusions, and in-stent restenosis [20]. The trial confirmed that OCT-guided PCI was non-inferior to IVUS in terms of device-oriented composite outcomes at one year, while offering more detailed lesion assessment and procedural standardization. This was echoed in the OCTOBER (Optical Coherence Tomography Optimized Bifurcation Event Reduction) trial, published in the New England Journal of Medicine, which specifically addressed bifurcation lesions—a scenario where precise wire positioning and stent expansion are critical. OCT guidance was associated with fewer unplanned side branch interventions, without compromising safety, and resulted in better overall procedural outcomes compared to angiography alone [21]. Adding further strength to the evidence base, the OCTIVUS (Optical Coherence Tomography versus Intravascular Ultrasound-Guided Percutaneous Coronary Intervention) trial—a large, multicenter, randomized non-inferiority study—compared OCT- and IVUS-guided PCI in over 2000 unselected patients. OCT proved non-inferior to IVUS in terms of major adverse cardiac events at one year, confirming its efficacy and safety in a broad clinical population [22]. Despite these strengths, OCTIVUS included a heterogeneous population of patients and lesion types, and the non-inferiority design limits conclusions about superiority. Additionally, procedural costs and contrast volume were higher in the OCT group, which may have implications in real-world practice, especially in patients at risk for contrast-induced nephropathy.

Crucially, a prespecified substudy of OCTIVUS focused specifically on complex coronary lesions, using a robust definition that included left main disease, bifurcations, aorto-ostial lesions, chronic total occlusions, long or heavily calcified lesions, in-stent restenosis, and multivessel disease [23]. In this subgroup, OCT guidance maintained non-inferiority to IVUS for the primary composite endpoint at a median follow-up of two years. Interestingly, despite higher contrast usage in the OCT group, there was no significant difference in contrast-induced nephropathy. Moreover, OCT-guided PCI was associated with significantly fewer procedural complications and lower rates of target vessel-related myocardial infarction, underscoring the clinical value of high-resolution imaging even in the most demanding cases.

Additionally, the FORZA trial (Fractional Flow Reserve or Optical Coherence Tomography to Guide Revascularization of Intermediate Coronary Stenoses) provided the first randomized head-to-head comparison between OCT and FFR guidance in patients with angiographically intermediate lesions [24]. At 13 months, OCT guidance was associated with a significantly lower rate of the composite endpoint of Major Adverse Cardiovascular Events (MACE) or significant residual angina, though at the cost of a higher number of PCIs and increased procedural expense. Notably, the 5-year follow-up confirmed comparable MACE rates between OCT and FFR groups, with similar or slightly lower rates of myocardial infarction and target vessel revascularization in the OCT arm, and no significant differences in all-cause mortality [25]. These findings support the notion that OCT guidance not only improves early clinical outcomes but also sustains long-term efficacy and safety, positioning it as a robust alternative to physiology-based strategies—especially in borderline or anatomically ambiguous lesions.

A growing body of evidence has positioned OCT as a central tool in contemporary PCI—not only for technical optimization but also for its unparalleled capacity to reveal the biological and structural complexity of coronary lesions. Unlike angiography or even physiological indices like FFR, OCT enables direct visualization of high-risk plaque features such as thin fibrous caps, lipid-rich cores, macrophage infiltration, and microcalcifications—parameters that reflect plaque vulnerability rather than hemodynamic severity.

This diagnostic power was highlighted in the COMBINE OCT–FFR trial [26], which prospectively assessed diabetic patients with intermediate, FFR-negative coronary lesions. OCT uncovered that a significant subset harbored thin-cap fibroatheromas (TCFAs), and strikingly, these patients experienced a fivefold higher rate of adverse cardiac events at 18 months compared to those without TCFA, despite having lesions deemed functionally non-significant. The central insight from COMBINE is clear: OCT reveals dimensions of coronary pathology that are invisible to physiology alone, suggesting that plaque biology may surpass ischemia as a driver of future events—especially in high-risk groups like diabetic patients. Nonetheless, this trial was observational in nature, and its findings—while provocative—remain hypothesis generating. The relatively small sample size and the absence of randomization limit causal inference. Moreover, the prognostic significance of OCT-detected TCFA, though compelling, has not yet been validated in large-scale prospective trials.

This reframing of risk assessment is being tested in the ongoing COMBINE-INTERVENE trial (NCT05333068), which seeks to determine whether OCT-guided identification and treatment of vulnerable plaques improves clinical outcomes beyond a physiology-guided strategy alone. The trial randomizes patients with multivessel disease to either FFR-only guidance or a hybrid OCT + FFR approach, and uniquely allows for focal stenting of high-risk plaques—even in the absence of ischemia. This approach marks a paradigm shift: from reactive treatment of flow-limiting lesions to proactive intervention based on vulnerable plaque morphology.

The clinical relevance of OCT-based insights is further underscored by the 2024 network meta-analysis by Stone et al. [27], which compiled data from over 15,000 patients across 22 randomized trials. The analysis demonstrated that intravascular imaging guidance—particularly with OCT or IVUS—was associated with substantial reductions in target lesion failure, cardiac death, myocardial infarction, and stent thrombosis, compared to angiography alone. Importantly, outcomes were equivalent between OCT and IVUS, reinforcing OCT’s role as a safe, effective, and more anatomically informative alternative.

Reflecting this expanding evidence base, the latest ESC guidelines have officially incorporated OCT into routine clinical recommendations. In chronic coronary syndromes (CCS), intravascular imaging (IVI)—including OCT—is now endorsed with Class I, Level A evidence for PCI guidance in complex anatomical scenarios such as left main disease, true bifurcations, and long lesions [28]. In acute coronary syndromes (ACS), IVI receives a Class IIa recommendation when the culprit lesion is angiographically apparent, and OCT specifically holds a Class IIb indication when the culprit is unclear or ambiguous [29]. These recommendations highlight the procedural and diagnostic value of OCT in enhancing lesion characterization, improving stent deployment, and reducing long-term adverse outcomes. In summary, as growing clinical trial evidence and a landmark meta-analysis converge, and with OCT now formally incorporated into international guidelines, its integration into standard interventional practice is no longer exploratory—it has become a cornerstone of precision-guided coronary care.

Despite robust evidence supporting the clinical utility of OCT, several limitations must be acknowledged to preserve a balanced perspective. First, its limited tissue penetration restricts full visualization of large, lipid-rich or heavily calcified plaques, potentially underestimating plaque burden. Additionally, OCT requires transient blood clearance using contrast media, making it less feasible in patients with renal dysfunction or hemodynamic instability, and potentially limiting its application in certain cases. Procedural cost and accessibility remain practical challenges. Compared to angiography or IVUS, OCT-guided interventions may incur higher costs, and image interpretation demands dedicated expertise. This steeper learning curve—both technical and interpretative—can impact adoption rates, particularly outside high-volume centers or in settings with limited intravascular imaging experience. Finally, although studies such as FORZA highlighted the added value of OCT in guiding PCI decisions, integration with functional assessments like FFR remains debated. These modalities provide distinct insights—anatomic versus physiologic—and ongoing trials, such as the COMBINE-INTERVENE study (NCT05333068), are exploring whether their synergistic use improves clinical outcomes or instead introduces unnecessary complexity and cost. Thus, while OCT significantly enhances the precision of coronary interventions, its optimal application requires consideration of both technical strengths and practical limitations. Judicious, patient-specific use remains essential.

## 6. From Treatment to Understanding: OCT as a Multifaceted Instrument of Clinical Guidance and Scientific Research

**Figure 3:**
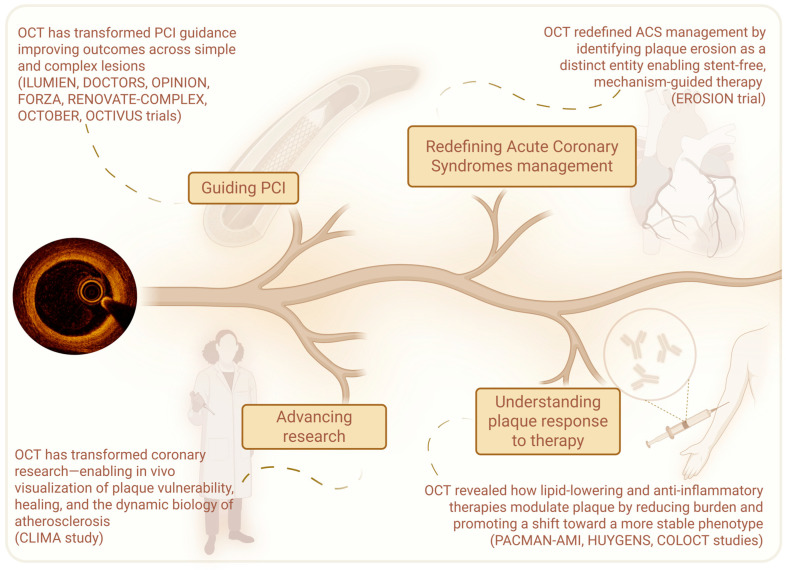
The evolving roles of OCT in contemporary cardiology: this schematic illustrates the multifaceted impact of OCT—from guiding PCI and redefining ACS treatment, to elucidating plaque biology, monitoring therapeutic effects, and advancing atherosclerosis research.

OCT has gone far beyond its initial role as a simple guide to PCI. It has transformed the way we approach ACS, shifting treatment from uniform protocols to mechanism-driven, lesion-specific strategies. At the same time, it has deepened our understanding of how therapies—particularly lipid-lowering and anti-inflammatory agents—modulate plaque characteristics, promoting a transition toward more stable phenotypes. Finally, its unparalleled resolution has enabled the in vivo investigation of coronary plaque biology with a level of detail previously reserved for histopathology. Through OCT, atherosclerosis is no longer viewed as a static disease but as a dynamic process marked by phases of vulnerability, rupture, healing, and progression. This shift has expanded our understanding of CAD, revealing the temporal and structural complexity of plaque behavior and offering new frameworks for risk assessment and therapeutic targeting (Figure 3).

### 6.1. Redefining Acute Coronary Syndromes: From Morphology to Management

The advent of OCT has redefined our approach to ACS, shifting the paradigm from uniform treatment strategies to lesion-specific, mechanism-guided management. One of the most transformative contributions of OCT has been the identification of plaque erosion (PE) as a distinct and relatively common substrate for ACS—particularly in younger patients, women, and smokers without classical cardiovascular risk factors [30]. Historically, PCI with stent implantation was considered standard care for virtually all ACS presentations. However, the landmark EROSION (Effective Anti-Thrombotic Therapy Without Stenting: Intravascular Optical Coherence Tomography-Based Management in Plaque Erosion) challenged this doctrine [31]. By utilizing OCT to differentiate between plaque rupture (PR) and PE in real time, it introduced the concept that not all ACS lesions necessitate stenting. Patients with preserved flow, non-obstructive lesions, and OCT-confirmed PE were treated conservatively with potent antithrombotic therapy alone—without stent implantation. The outcomes were compelling: over 90% of patients demonstrated thrombus regression and plaque healing on follow-up imaging, with a low incidence of major adverse cardiac events (MACE). This study catalyzed a broader reevaluation of ACS management, highlighting the importance of matching therapy to plaque biology rather than relying solely on angiographic or clinical criteria. OCT enabled direct visualization of intact fibrous caps, superficial thrombus, and the absence of deep plaque cavities—hallmarks of erosion—thus allowing physicians to identify lesions amenable to pharmacologic stabilization. The implications of this shift are profound. In selected cases, a stent-free strategy may reduce the risk of late thrombosis, eliminate the need for prolonged dual antiplatelet therapy, and promote natural endothelial healing. Moreover, follow-up data from longer-term cohorts have reinforced the safety of this approach, particularly when early thrombus resolution is observed—serving both as a marker of therapeutic success and a predictor of favorable prognosis [32,33]. In conclusion, by enabling in vivo assessment of plaque morphology with exceptional resolution, OCT has emerged as a key enabler of personalized cardiology—allowing clinicians to move beyond uniform treatment algorithms and instead align therapy with the distinct structural and biological characteristics of each lesion. In this way, OCT not only enhances therapeutic precision but also lays the groundwork for mechanism-driven, individualized care in the management of ACS.

### 6.2. Understanding Plaque Response to Therapy in Atherosclerosis: OCT as a Potential Biological Marker

Beyond its interventional applications, OCT has become an indispensable tool for observing the temporal evolution of CAD and for assessing how therapeutic strategies—particularly lipid-lowering therapies (LLT)—alter plaque composition and stability over time. By offering near-histological resolution in vivo, OCT enables dynamic monitoring of atherosclerotic lesions, capturing subtle changes in fibrous cap thickness, lipid burden, and inflammatory features. This capability has transformed our understanding of how medical therapies modulate disease progression, positioning OCT at the forefront of research into the mechanisms of plaque stabilization and regression.

Current guidelines emphasize aggressive lipid management after an ACS, underscoring the importance of early and intensive LDL-C reduction. The European Society of Cardiology now recommends achieving LDL-C levels below 1.4 mmol/L (55 mg/dL), or even <1.0 mmol/L (40 mg/dL) in patients with recurrent events [28]. This is to be accomplished through high-dose statins, with the early addition of ezetimibe and Proprotein Convertase Subtilisin/Kexin Type 9 (PCSK9) inhibitors in those not reaching lipid goals.

It is now well established that LLT, by reducing circulating LDL-C levels, significantly decrease cardiovascular events—a finding consistently demonstrated across multiple large-scale clinical trials [34,35,36,37,38,39]. Among these, the MIRACL trial [34] established the superiority of atorvastatin 80 mg daily over placebo in patients with recent ACS, demonstrating a significant reduction in early recurrent ischemic events within just 16 weeks of therapy initiation. Building on this, the PROVE-IT TIMI 22 trial [35] confirmed the clinical benefit of intensive lipid lowering by showing that atorvastatin 80 mg was superior to pravastatin 40 mg in reducing major adverse cardiovascular events in post-ACS patients. Together, these two pivotal studies laid the foundation for current guidelines recommending early and intensive statin therapy as a cornerstone of secondary prevention following ACS, shaping contemporary lipid-lowering strategies in high-risk cardiovascular patients.

However, OCT has provided a deeper understanding of how these therapies exert their benefit. Beyond simply reducing plaque burden, LLT has been shown to induce meaningful changes in plaque morphology. OCT imaging has revealed that such treatments promote favorable morphological changes—thickening of the fibrous cap, reduction in lipid core size, and attenuation of inflammatory features—effectively guiding plaques toward a more stable, less rupture-prone phenotype. This mechanistic insight underscores the fact that achieving lipid targets is not merely a biochemical goal, but a means to directly modulate plaque vulnerability and, ultimately, clinical risk.

The impact of intensive LLT on both plaque burden and phenotype is well established through multiple landmark IVI trials. The REVERSAL (Reversal of Atherosclerosis with Aggressive Lipid Lowering) trial, using IVUS, demonstrated that high-intensity statin therapy (atorvastatin 80 mg) halted the progression of coronary atherosclerosis more effectively than moderate-intensity statin therapy (pravastatin 40 mg), with superior reductions in LDL-C and inflammatory markers [40]. Building on this, the ASTEROID (A Study to Evaluate the Effect of Rosuvastatin on Intravascular Ultrasound-Derived Coronary Atheroma Burden) study provided the first robust intravascular evidence that intensive statin therapy not only stabilizes but can actually induce regression of coronary atherosclerosis. In this open-label, prospective trial, 507 patients with established CAD received high-intensity rosuvastatin (40 mg/day), achieving unprecedented reductions in LDL-C (mean 61.1 mg/dL) and a significant increase in HDL-C. Intravascular ultrasound performed at baseline and after 24 months revealed a statistically significant decrease in percent atheroma volume (PAV) and total atheroma volume (TAV). These findings demonstrated that very aggressive LDL-C lowering can induce regression of atherosclerotic plaque, and helped establish the clinical rationale for intensive LLT therapy in both chronic and acute settings [41]. The YELLOW (Reduction in yellow plaque by aggressive lipid-lowering therapy) trial added further insight by demonstrating that even short-term intensive statin therapy could reduce lipid content within coronary plaques, as measured by near-infrared spectroscopy (NIRS), suggesting a rapid impact on plaque composition in patients with obstructive coronary artery disease [42]. Expanding beyond statins, the GLAGOV (Effect of Evolocumab on Progression of Coronary Disease in Statin-Treated Patients) [43] trial investigated the addition of the PCSK9 inhibitor evolocumab to background statin therapy. IVUS imaging revealed that this combination not only achieved deeper LDL-C reduction but also led to greater plaque regression compared with statins alone—highlighting the incremental anatomical benefits of targeting PCSK9. However, these findings were largely based on IVUS and NIRS technologies, which offered volumetric or compositional data but lacked the resolution to interrogate microstructural features.

It was OCT that finally made it possible to see the biology of healing unfold. In the HUYGENS (High-Resolution Assessment of Coronary Plaques in a Global Evolocumab Randomized Study) [44] trial, patients with non-ST elevation ACS were treated with evolocumab on top of high-intensity statins and underwent serial OCT imaging. After 52 weeks, evolocumab therapy resulted in a significant increase in minimum fibrous cap thickness, reduction in lipid arc, and a decrease in macrophage presence, indicating a substantial stabilization of high-risk plaques. These findings were corroborated by the PACMAN-AMI trial, which enrolled patients with recent ST-elevation myocardial infarction and randomized them to receive alirocumab or placebo on top of statin therapy. After 52 weeks, OCT imaging of non-culprit plaques showed that alirocumab significantly increased fibrous cap thickness compared to placebo and produced a greater reduction in maximum lipid arc. These changes were consistent across multiple coronary segments and were supported by complementary imaging (IVUS and NIRS) that demonstrated reductions in total atheroma volume and lipid burden [45].

Taken together, these OCT-based trials confirm that intensive LLTs not only reduce systemic LDL-C levels but also directly modify plaque morphology—promoting cap thickening, lipid core reduction, and inflammatory resolution. This morphological transformation underpins the transition to a more stable plaque phenotype, offering a mechanistic explanation for the clinical benefits observed in outcome-driven trials. Moreover, these trials confirm OCT’s capacity to quantify the effects of LLT at the tissue level—offering not just surrogate markers of efficacy, but direct morphologic endpoints. Despite the strength of these data, current clinical practice does not yet incorporate plaque imaging into the algorithm for escalating LLT. Nonetheless, OCT may serve in the near future as a decision-making tool to personalize treatment in patients with residual biological risk, despite optimal pharmacologic management.

In parallel, inflammation has gained recognition as a key contributor to atherosclerotic progression and plaque destabilization. Elevated inflammatory markers, such as high-sensitivity C-reactive protein, are associated with both disease severity and event recurrence, independent of LDL levels [46,47,48]. Given this growing understanding, several studies have explored whether directly targeting inflammation—independent of lipid levels—could translate into improved cardiovascular outcomes. This has led to the investigation of anti-inflammatory therapies as a complementary strategy to conventional lipid-lowering approaches in the prevention of ischemic events.

The CANTOS (The Canakinumab Anti-inflammatory Thrombosis Outcome Study) trial demonstrated that targeting interleukin-1β with canakinumab reduced MACE independently of LDL-C levels [49]. The COLCOT (Colchicine Cardiovascular Outcomes Trial) [50] and LoDoCo2 (Low-Dose Colchicine 2) [51] trials later confirmed the efficacy of colchicine in both ACS and CCS populations, providing compelling support for inflammation-targeted strategies in patients already on optimal statin therapy. The COLOCT (Colchicine–Optical Coherence Tomography Trial) [52] study bridged the systemic and local perspectives by evaluating the impact of colchicine on plaque characteristics using OCT. In a randomized cohort of ACS patients with lipid-rich plaques, those receiving colchicine exhibited significantly greater increases in fibrous cap thickness and reductions in lipid arc and macrophage infiltration than those on placebo. These morphologic changes were accompanied by reductions in systemic inflammatory biomarkers, suggesting a concordant biologic effect at both vascular and systemic levels. This convergence of systemic and plaque-specific data positions OCT as a potentially powerful tool for guiding anti-inflammatory therapies in the future. By identifying high-risk plaque features and monitoring their modification under treatment, OCT could help target patients most likely to benefit from emerging therapeutic strategies—whether lipid-lowering, anti-inflammatory, or both.

Although no formal recommendations currently exist for adjusting therapy based on plaque phenotype, the groundwork has been laid. OCT provides a rare window into the biology of atherosclerosis in real time, making visible the silent healing—or progression—of the disease. As trials like PACMAN-AMI, HUYGENS, and COLOCT continue to shape our understanding, OCT may soon evolve from a diagnostic device to a true biological compass, orienting treatment around the tissue-level realities of coronary disease.

### 6.3. OCT as a Scientific Tool: Exploring the Dynamic Behavior of Atherosclerosis

Beyond its clinical applications, OCT has established itself as a cornerstone in cardiovascular research, offering unique insights into the dynamic nature of coronary atherosclerosis—particularly in advancing our understanding of plaque vulnerability and the mechanisms of plaque healing.

The concept of plaque vulnerability has long been at the heart of research, dating back to seminal histopathological studies on victims of sudden cardiac death. These early investigations consistently identified a recurrent morphological pattern: plaques capped by a thin fibrous layer and marked by a large necrotic core. These so-called TCFAs were recognized as highly rupture-prone lesions capable of triggering thrombosis following fibrous cap disruption and subsequent exposure of thrombogenic material to the bloodstream [53]. With the advent of IVI—and in particular, OCT—it became possible to identify these features in vivo, rather than relying on post-mortem analysis. OCT, with its near-microscopic resolution, has emerged as the reference standard for detecting TCFAs in living patients, allowing detailed visualization of fibrous cap thickness, lipid core dimensions, and other subtle features of plaque vulnerability [54], including macrophage infiltration [55], cholesterol crystals [56,57], and intraplaque microchannels [58]—which reflect active inflammation.

OCT was instrumental in establishing the clinical relevance of TCFAs as the morphological substrate of rupture-prone plaques. A wealth of OCT-based studies has since confirmed that TCFAs are significantly more prevalent in patients presenting with ACS compared to those with stable disease, and are also more frequently observed in patients experiencing recurrent cardiovascular events [26,58,59,60,61].

A robust body of evidence from IVI studies has clarified the link between plaque characteristics and the risk of future coronary events. In the PROSPECT (The Providing Regional Observations to Study Predictors of Events in the Coronary Tree) study [62], three-vessel IVUS and virtual histology (VH-IVUS) were used to assess non-culprit lesions in patients with ACS. The study found that plaques with a burden >70%, small minimal lumen area (<4.0 mm^2^), and TCFA morphology were independently associated with a higher rate of MACE at three-year follow-up. PROSPECT II, using a combination of IVUS and NIRS, confirmed these findings and highlighted that non-culprit lesions rich in lipid (as detected by high LCBI values on NIRS) also carried elevated risk—even in angiographically mild disease [63].

The VIVA (VH-IVUS in Vulnerable Atherosclerosis) [64] study, based on VH-IVUS imaging, further emphasized the prognostic significance of plaque burden. It showed that lesions with a plaque burden >70% were associated with a significantly higher risk of MACE at 12 months, particularly when presenting with fibroatheroma features. Similarly, the LRP (lipid-rich plaque) [65] study employed NIRS-IVUS to quantify lipid content across coronary lesions. The trial demonstrated that plaques with a maximal lipid-core burden index over 4 mm (maxLCBI_4_mm) >400 were strongly predictive of future coronary events over a two-year follow-up, underscoring the role of lipid-rich cores in plaque instability. Finally, the CLIMA study [66]—using OCT—offered the highest spatial resolution to date in assessing plaque vulnerability. Conducted in over 1000 patients undergoing coronary angiography, it identified four high-risk features associated with future cardiovascular death and target-vessel myocardial infarction: TCFA (cap thickness <75 μm), lipid arc > 180°, macrophage accumulation, and minimal lumen area <3.5 mm^2^.

Taken together, these findings support a dual model of vulnerability: one based on plaque phenotype, particularly the presence of TCFA and associated high-risk features, and one grounded in the overall burden of disease. The convergence of these two dimensions—morphology and extent—offers a robust framework for identifying patients at increased risk and underscores the unique role of OCT in advancing the field from histology to precision imaging. Moreover, OCT has not only clarified the morphological traits of high-risk plaques but also revealed that vulnerability is not confined to TCFAs. Increasing evidence has shown that PE and eruptive-calcified nodules also represent triggers of ACS, each with distinct structural and cellular profiles. This has led to a broader, more inclusive definition of “vulnerable plaques” encompassing all major substrates capable of inducing thrombosis [67].

In research contexts, equally transformative has been the ability of OCT to capture healed plaques. The concept of plaque healing—previously confined to post-mortem histological observations—was brought to the forefront by a pivotal review published in The New England Journal of Medicine in 2020 by Rocco Vergallo and Filippo Crea [68]. In that work, the authors proposed a dynamic interpretation of atherosclerosis, highlighting that plaque rupture is not always a clinically manifest event: in many cases, it may be silent and undergo spontaneous healing. OCT has been instrumental in bringing this concept into the clinical setting, enabling in vivo identification of healed plaques by revealing layered structures within the intima—histologically consistent with cycles of rupture and subsequent repair [69,70]. While initial OCT studies linked healed plaques predominantly to patients with CCS [61], subsequent research in the setting of ACS has reshaped this view. Several analyses have shown that healed morphologies—typically characterized by layered structures—are also common in non-culprit lesions of patients with acute presentations. Interestingly, these plaques often share features of vulnerability with culprit sites, including lipid-rich cores, and macrophage infiltration [69,71]. This convergence of findings has prompted a shift in perspective: healed plaques should no longer be considered exclusive markers of stability, but rather footprints of previous plaque destabilization—evidence of a coronary environment prone to rupture and thrombosis. A serial OCT study has also helped clarify the implications of this phenomenon. Non culprit lesions developing new layered patterns over time have been associated with features of previous vulnerability, including TCFA, inflammation, and thrombosis. Over follow-up, these plaques tend to show cap thickening and lipid depletion—indicating repair—but also progressive luminal narrowing, which may ultimately increase the risk of future ischemic events [72].

Despite these advances, many questions remain. As Vergallo and Crea pointed out, we still lack reliable predictors of which plaques will heal silently versus those that will progress to clinical events. Why do some individuals undergo efficient healing, while others do not? [68]

To address these unanswered questions, our research group has launched a prospective OCT-based study: the VISION study (EValuating the role of IGF-1 and S-Klotho In plaque phenOtype and vulNerability; NCT06522074). This trial explores the relationship between plaque characteristics—vulnerability and healing—and the growth hormone/insulin-like growth factor 1–soluble Klotho axis, which has been implicated in vascular homeostasis and inflammation. By integrating OCT with molecular profiling, VISION aims to unravel the biological determinants that influence plaque healing shedding light on one of the most elusive mechanisms in CAD.

Thus, OCT has reoriented coronary research from luminal geometry to tissue biology, from single time-point measurements to temporal dynamics, and from speculation to direct visualization. It has become, quite literally, a lens through which the unseen processes of CAD can finally be studied in living patients.

## 7. Contemporary Advances: Artificial Intelligence, Micro-OCT, and Hybrid Imaging (2010–Today)

Since its clinical consolidation in 2010, OCT technology has undergone continuous refinement. Rather than remaining static, it has evolved in resolution, functionality, and integration into cardiovascular diagnostics and interventional practice—expanding its utility across previously unaddressed clinical scenarios.

### 7.1. From Data to Diagnosis: The Rise of Artificial Intelligence in OCT

With tens of thousands of scans now produced in cardiology and ophthalmology each year, the burden of interpretation has shifted from expert alone to expert-plus-machine. Artificial intelligence (AI) is now increasingly deployed to assist in identifying lesions, segmenting anatomical features, and supporting procedural decisions. These technologies help minimize inter-observer variability and enhance diagnostic reliability. A prime example is the Ultreon™ 2.0 software platform (Abbott, TX, USA), which combines OCT and angiographic imaging with AI-driven analytics. Designed to integrate seamlessly into catheterization lab workflows, Ultreon™ 2.0 automatically identifies key vessel features—such as lumen size, calcium distribution, and stent positioning—and delivers real-time guidance for PCI optimization. Its intuitive interface and immediate feedback allow for more informed decisions directly at the point of care. Looking ahead, platforms like this could pave the way for fully automated lesion assessment, earlier detection of high-risk plaques, and predictive modeling based on large-scale imaging data.

### 7.2. Micro-OCT: Toward Cellular Imaging

Meanwhile, in the research realm, micro-OCT (μOCT) pushes the resolution boundary into the submicron range. Imaging at \~1 μm resolution, μOCT allows for visualization of macrophage clusters, endothelial cells, and intimal microstructures in atherosclerosis—essentially transforming the catheter into a live, in situ microscope [73]. Although still investigational, μOCT represents a bridge toward histology-like fidelity without biopsy. Its promise lies especially in identifying vulnerable plaques and inflammatory activity at the cellular level, potentially guiding early preventive strategies in cardiovascular medicine.

### 7.3. The Hybrid Path: OCT–IVUS Integration

The clinical landscape of IVI is rapidly evolving with the emergence of hybrid IVUS–OCT systems, which unify the high-resolution benefits of OCT with the deeper tissue penetration of IVUS. Several devices are now commercially available or in advanced clinical development. Terumo Corporation (Tokyo, Japan) has developed a system that enables reconstruction of co-registered images from both OCT and IVUS, allowing enhanced assessment of vessel architecture across all layers. Similarly, the Novasight Hybrid system by Conavi Medical Inc. (Toronto, ON, Canada) offers simultaneous acquisition of OCT and IVUS cross-sectional images from nearly identical locations, ensuring spatial alignment between the two modalities during pullback. However, both platforms currently lack functionality for integrated physiological assessment, such as IVI-based FFR evaluation. A more comprehensive solution is represented by the PANOVISION system, developed by PANOVISION Co., Ltd. (Hangzhou, China), which integrates real-time co-registered OCT and IVUS imaging with extended pullback capabilities (up to 150 mm) and higher speeds (up to 40 mm/s) [74].

The first-in-human PANOVISION trial, a multicenter, randomized, open-label study, confirmed the system’s clinical robustness and non-inferiority to standalone IVUS or OCT. It demonstrated high imaging clarity, efficient complication detection post-PCI (e.g., tissue prolapse and malapposition), and no device-related adverse events [75]. Similarly, the SUPERIOR study [76] provided multicenter data on the Novasight Hybrid system, validating its safety and technical performance in over 110 patients undergoing PCI in China. The system achieved a 99.1% device success rate, 98.2% technical success, and over 96% of imaging segments met predefined clarity criteria. Notably, no catheter-related major adverse events occurred. The study emphasized the system’s versatility, with potential applications ranging from morphological characterization of high-risk plaques to optimizing stent deployment and evaluating therapeutic response.

Collectively, these hybrid platforms signify a paradigm shift in IVI, moving toward all-in-one systems capable of delivering anatomical, structural, and potentially functional insights in a single pass. As integration with physiology and AI-assisted analytics progresses, hybrid IVUS–OCT systems are poised to redefine precision imaging strategies in coronary interventions.

### 7.4. Contrast-Free OCT: A Feasible and Accurate Imaging Alternative

A commonly cited limitation of OCT in clinical practice is the requirement for iodinated contrast media to displace intraluminal blood and ensure adequate image quality. This can be particularly concerning in patients with pre-existing renal dysfunction, where the risk of contrast-induced nephropathy may prompt operators to avoid OCT in favor of alternative modalities such as IVUS. However, growing evidence suggests that this limitation may be more manageable than previously assumed. Experimental and clinical studies have explored the use of alternative flushing solutions, such as normal saline or diluted contrast, to perform OCT imaging without compromising diagnostic utility.

A controlled preclinical and clinical investigation showed that saline-based OCT imaging, while producing slightly smaller vessel dimensions compared to contrast, demonstrated a strong linear correlation that allowed for the application of correction equations. After adjustment, image interpretation—including lumen measurements and stent strut resolution—remained consistent with contrast-based OCT [77,78]. In a prospective clinical comparison, saline and contrast OCT runs performed at identical anatomical sites showed no statistically significant differences in key quantitative parameters such as minimal lumen area, proximal and distal reference diameters, or percentage area stenosis. Importantly, plaque morphology and stent apposition were equally well visualized, with high image clarity in both modalities [77,79].

Taken together, these data suggest that a contrast-free or contrast-minimized approach to OCT is not only technically feasible but also diagnostically robust. While further validation in larger cohorts is warranted, this evolving technique may allow broader use of OCT in patients with renal impairment, particularly when combined with strategies to limit overall contrast exposure. As a further consideration, OCT-guided PCI has been shown to reduce the number of angiographic acquisitions typically required during the procedure, which may help offset contrast use overall—making its use in selected patients with renal dysfunction a reasonable and increasingly viable option.

As a further consideration, OCT-guided PCI can allow for a significant reduction in angiographic projections during the procedure—and consequently, in the use of contrast media. Operators can rely on the OCT pullback for both lesion assessment and post-PCI result evaluation, often requiring only a single angiographic view to acquire the imaging run. This approach enables precise planning and optimization while minimizing contrast exposure, offering a streamlined and contrast-sparing workflow.

## 8. Epilogue—Prometheus Redux

OCT’s journey is a modern echo of the Promethean gift. Once confined to the eye, then adapted to the heart, this flame has grown stronger with every iteration. Yet, Prometheus’s tale warns us that power demands responsibility. As AI-guided diagnosis, hybrid devices, and spectral imaging proliferate, we must remain vigilant: in validation, in equity of access, and in ethical deployment. But if used wisely, this flame will not only reveal disease. It will guide the future of cardiovascular medicine itself.

As we reach the end of this historical review, it is worth turning to a phrase that has become emblematic of scientific progress. In a letter dated 5 February 1675, addressed to his rival Robert Hooke, Isaac Newton wrote: “If I have seen further, it is by standing on the shoulders of giants.” While often cited as a mark of humility, this phrase also carries deeper historical resonance. Newton was likely referencing an earlier formulation attributed to Bernard of Chartres, a 12th-century philosopher, who, according to John of Salisbury, used to say: “We are like dwarfs on the shoulders of giants, so that we can see more and farther than they.” The metaphor reflects the idea that innovation does not occur in isolation, but rather builds on a foundation laid by predecessors—each generation of thinkers enabling the next to see a bit further. In the context of Newton’s correspondence, the remark may have also held a complex tone: Robert Hooke, while a brilliant experimentalist, had a famously tense relationship with Newton. Some historians speculate that the “giants” in Newton’s sentence may have been a subtle exclusion of Hooke himself—who was notably short and physically deformed—though this interpretation remains debated. Regardless of any personal undertones, the quote has endured because it captures a universal truth: that scientific advancement is inherently cumulative.

This perspective is especially relevant in the story of OCT. From Michel Duguay’s early insight that light might probe biological tissue, to Fercher’s work on low-coherence interferometry, to the MIT team’s realization of clinical applications, each step was dependent on the groundwork laid before it. Acknowledging this lineage is more than an academic formality—it provides essential context for understanding the capabilities, limitations, and future potential of the technology. In medicine and engineering alike, tracing the origins of our tools reminds us that today’s breakthroughs rest on the layered insights of generations past. By recognizing the history behind OCT, we not only honor its pioneers—we equip ourselves to push its boundaries further still.

## Data Availability

All data generated or analyzed during this study are included in this published article. Further inquiries should be directed to the corresponding author.

## Abbreviations

The following abbreviations are used in this manuscript:

OCT	Optical coherence tomography
CAD	Coronary artery disease
PCI	Percutaneous Coronary Intervention
PCI	Partial Coherence Interferometry
MIT	Massachusetts Institute of Technology
TD-OCT	Time-domain OCT
FD-OCT	Frequency-domain OCT
IVUS	Intravascular ultrasound
FDA	Food and Drug Administration
MGH	Massachusetts General Hospital
FFR	Fractional Flow Reserve
TCFA	Thin-cap fibroatheroma
CCS	Chronic coronary syndrome
IVI	Intravascular imaging
ACS	Acute coronary syndrome
PE	Plaque erosion
PR	Plaque rupture
MACE	Major adverse cardiac events
LLT	Lipid-lowering therapies
PCSK9	Proprotein Convertase Subtilisin/Kexin Type 9
NIRS	Near-infrared spectroscopy
VH-IVUS	Virtual histology intravascular ultrasound
AI	Artificial intelligence
μOCT	Micro-OCT
